# Perception, risk factors, and health behaviours in adult obesity in Kolkata, India: a mixed methods approach

**DOI:** 10.1186/s12889-022-14531-9

**Published:** 2022-12-19

**Authors:** Somdutta Barua, Nandita Saikia

**Affiliations:** 1 Research Scholar, Centre for the Study of Regional Development, School of Social Sciences, Jawaharlal Nehru University, New Delhi, India; 2 Research Associate, National Institute of Urban Affairs (NIUA), New Delhi, India; 3 Professor, Department of Public Health and Mortality Studies, International Institute for Population Sciences, Mumbai, India

**Keywords:** Risk factors, Diet, Physical activity, Obesity, Perception, India

## Abstract

**Background:**

World Health Organisation has described obesity as one of the most neglected public health issues. Initially, obesity was only a problem in high-income countries; however, at present, it is rising in middle and low-income countries as well, rapidly in India, especially in the urban areas. In the light of the increasing prevalence of obesity in India, it was worthwhile to study perception, risk factors and health behaviours in adult obesity holistically.

**Methods:**

This study resorted to a concurrent mixed methods approach, collecting and combining quantitative survey (*n* = 120) and qualitative interview data (*n* = 18). Female and male aged 25–54 years with a waist circumference of 80 cm and 90 cm or higher, respectively, and a BMI of 25 or higher were selected from Kolkata, India. Kolkata was chosen as the study area since it ranked 7th out of 640 districts, the highest among the five major urban cities in India, with around 41% of the female and 43% of the male population aged 15–49 years with a BMI of 25 or higher.

**Results:**

Participants confirmed that lifestyle was one of the main reasons for obesity. They believed that family history, social relations, behavioural factors, urbanisation, and time-poor were significant risk factors of obesity. Interview participants expanded that technology, lack of health education and self-care, and digital marketing of food influenced the risk of obesity. Participants confirmed that they wanted to lose weight to feel healthier. Most respondents claimed that they engaged in lightly to moderate-intensity physical activity. However, a discrepancy in opinion was observed between survey responses and interview participants’ views on dietary behaviours. Participants confirmed that they rarely consulted health professionals and that the family had a minimal role in preventing obesity. Interview participants expanded that people should make better lifestyle choices at an individual level to prevent obesity.

**Conclusions:**

Health education is fundamental. Making better lifestyle choices is crucial, which would help increase the lifespan and health span and decrease the risk of diseases. In addition, social support and better policies are required to prevent the disease and any related complications.

**Supplementary Information:**

The online version contains supplementary material available at 10.1186/s12889-022-14531-9.

## Introduction

World Health Organisation (WHO) has described obesity as one of the most neglected public health issues [1]. The increasing obesity prevalence creates a massive threat to public health. It increases exposure to other non-communicable diseases [2, 3] and reduces total lifespan and health span. Initially, obesity was only a problem in the United States and other high-income countries; however, at present, it is rising in middle and low-income countries as well, rapidly in India and China, especially in the urban areas [4].

A perception-based study on 15 adolescents with excess weight claimed obesity as a disease [5]. Factors like family history [6–9], the family’s eating habits [9], fatty foods [10], sleep [8, 10, 11], stress [11–13], more hours of sedentary activities [14, 15]; advertising [16, 17] and urbanisation [18] are known to influence the risk of obesity. Diet and physical activity are the two significant factors that could overpower one’s deep-seated physiology to maintain a balance of energy [19]. Prior studies observed that individuals with excess weight intended to participate in behaviour change and aimed to lose weight [20, 21] by exercising [20] and/or eating a balanced diet [20] or both [5].

Research suggests that a low carbohydrate diet, usually high in protein, supports weight loss. Protein helps regulate body weight as higher protein intake has a greater thermic effect, promotes greater satiety, and reduces overall calorie intake. Whole grains and high-fibre foods also promote higher satiety, thus reducing energy intake. On the other hand, studies have found that sugar-sweetened beverages promote weight gain and obesity. Besides, heavy alcohol consumption may lead to health problems and weight gain. Schulze and others studied the cohort of 51,603 women aged 26–46 from 1991 to 1999. Women who followed a western diet consisting of a higher portion of processed and refined foods and potatoes had the maximum weight gain. In contrast, women on a prudent diet with higher consumption of vegetables, fruits, fish, poultry and whole grains had the least [4].

Globally, obesity literature finds increased attention on soft drinks and fast-food consumption [22], especially since, in general, people tend to overeat them for being pleasurable to their taste buds. Attachment to food is usually emotional, especially for the depressed, and thus, a powerful incentive is required to break through the emotional bond. Furthermore, people of all educational levels and ages make bad food choices, consuming fewer vegetables and fruits while becoming more sedentary. Occupations requiring physical work are becoming bygones as more people are involved in service-sector jobs that are primarily sedentary [10]. Moreover, findings from many studies reveal that increased physical activity hinders gaining waist circumference and weight. In combination with dietary intervention, physical exercise has a more significant effect on weight management [4]. On the contrary, people are highly dependent on automobiles in modern times, choosing the elevator over the stairs even for two floors and driving for a short distance [9]. That is how lifestyle influences the cost and burden of medical facilities [10].

While India has already been battling undernutrition, the country is now facing another malnutrition problem, i.e., obesity [23]. According to NFHS 2015–16, India has about 20% of the population aged 15–49 years with a BMI of 25 or higher, with 241 out of 640 districts having a higher prevalence than the national average [24] and much higher in the urban counterparts than rural [23]. Obesity/overweight was significantly associated with a family history of obesity and thyroid problems in a recent Tamil Nadu study. With rapid urbanisation, including junk food in the diet and little physical work in daily routine, has also increased BMI levels among rural residents [7]. Findings from a cross-sectional study based on schools in Udaipur city of Rajasthan, revealed that most children with excess weight had a family history of obesity and slept less than 8 hours a day [8]. Another school-based study in Kolkata explained that the higher prevalence resulted from low awareness regarding healthy food habits and poor diet [25].

Yet, there is a dearth of studies on India related to these issues. Therefore, in-detailed studies are needed for timely and crafted policy intervention. Since the prevalence of diseases is rising, it is worthwhile to study perception, risk factors and health behaviours among adults with excess weight, which could help design necessary interventions and programs for public well-being. We found the participatory/advocacy philosophical worldview appropriate for the research problem. Furthermore, using mixed methods, no previous studies have explored the perception, risk factors, and health behaviours among adults with excess weight in the Kolkata metropolitan area. Quantitative surveys helped get a numeric description of the perception and behaviours. On the other hand, qualitative interviews helped corroborate and further examine the respondents’ actual insights [6]. The present study’s novelty is that the study is based on the recently collected qualitative and quantitative field data. The principal investigator (PI) measured each participant’s height, weight, and waist circumference.

## Methods

### Research design

**Fig. 1:**
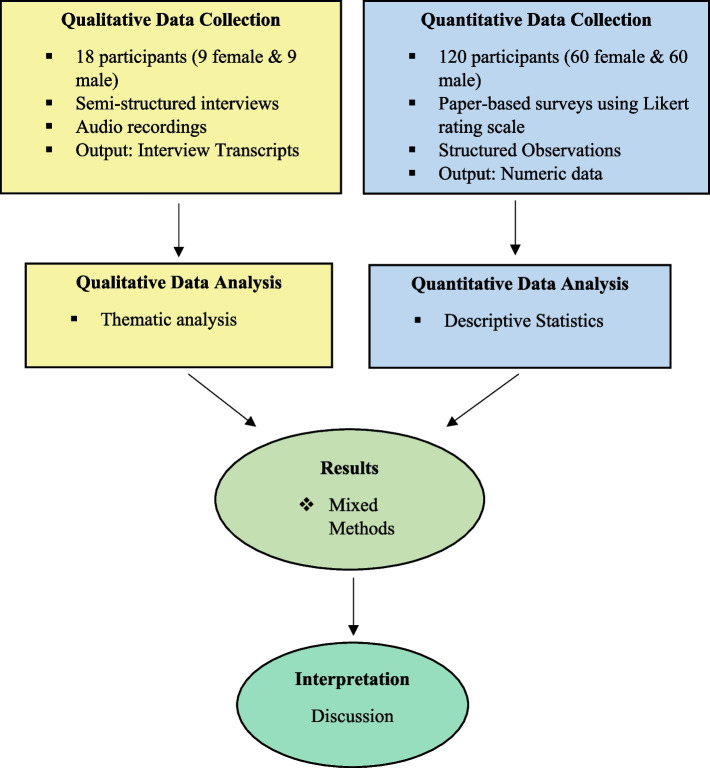
Diagram of Concurrent Mixed Methods Design (Forman, 2019)

We resorted to a concurrent mixed methods design, i.e., quantitative and qualitative data were collected simultaneously at the same field visit [26]. We separately gathered and analysed quantitative (closed-ended) and qualitative (open-ended) data before combining them. We placed equal importance on quantitative numbers and qualitative stories [26, 27]. We opted for separate sampling, meaning quantitative and qualitative samples were different. In Fig. 1, we provided a diagram of convergent mixed methods design. To design the framework and field instruments for this study, we considered the Health Belief Model and the Social-Ecological Model. These models and the chosen philosophical worldview assumptions helped design and direct the study to make it more participatory and change-oriented.

### Sampling, study population and data collection procedure

Kolkata city area was chosen as the study area since the Kolkata district ranked 7th out of 640 districts, the highest among the five major urban cities in India, with around 41% of female and 43% of male population aged 15–49 years had a BMI of 25 or higher and a mean BMI of about 23 [24]. We used purposive and snowball nonprobability sampling for the qualitative and quantitative parts of this investigation. We chose purposive sampling because we planned to study females and males aged 25 to 54 years who had a waist circumference of 80 cm and 90 cm, respectively, had a BMI of 25 or above, and lived in the Kolkata metropolitan region for the previous 6 months because it  was plausible that the person who had recently moved into the city area may not share the same perception or understanding on excess weight or similar health behaviours. We chose this age group and people with excess weight because, firstly, Hu [4] stated that increasing age tended to make people gain weight, especially during their mid-age. Secondly, this chosen group represented the most productive years of an individual’s life. During this period, careers are established, marriage takes place, and children are reared. Therefore, it is crucial to prioritise lifestyle patterns and set up preventive health patterns for self and family members. Last but not least, prior literature has found that people with excess weight are more susceptible to sociological and clinical complications. Further, we chose snowball sampling because there was no data to identify the targeted study population. Secondly, household or door-to-door survey was not chosen due to the sensitivity of this topic because of the probable stigma attached to bigger bodies.

Individuals were contacted via phone or social media, and the remaining participants were recruited with the help of other research participants. The survey and interview participants were pre-informed about the purpose and methodology of this study, and the PI obtained their approval via a signature on a consent form. The PI informed the interviewees in advance that their discussions would be audio-recorded. Detailed information was given about the study’s benefits, repercussions, anonymity, and confidentiality of the participants and their rights; participants’ questions were also answered regarding the study context. No individual was forced to participate in the study or penalised for non-participation. Only one available member from each household was considered. The PI used a standard long tape, a weighing machine, and a waist measuring tape to measure each participant’s height, weight, and waist circumference. Depending on the respondents’ preferences, quantitative questionnaires were given out to be filled out by the participants or communicated by the PI during the fill-out. Interviews were conducted at per participants’ convenient time and places. The PI completed eighteen interviews until she reached saturation; hence when the PI was ensured that there was just enough data, she ended the qualitative interviews. During the surveys and interviews, the PI tried to be respectful, unbiased, harmonic, and non-judgmental as much as possible. To uphold the study’s ethics, the final results have been presented truthfully [28].

### Regulatory and ethical aspects

The Institutional Ethics Review Board of Jawaharlal Nehru University (IERB-JNU) gave the approval for collecting data and approved the study protocol. Written consent was obtained from each participant before the survey and the interview. All data are kept anonymous and confidential.

### Instruments used for data collection

Prior to the fieldwork, the questionnaires were presented to a group of research scholars and an expert faculty member at Jawaharlal Nehru University and modified according to the suggestions received. Further, the survey questionnaire pre-testing was conducted with 6 participants, followed by a pilot survey with 40 participants. On the other hand, the interview guide was pre-tested with 3 participants, followed by a pilot survey with 6 participants. Individual participants were asked: a) what do you understand from this question; b) could this question be asked more simply; c) what is the meaning of this question; d) is the response choices understandable, or would you be like the format choices to be simpler; e) how did you find the survey; f) did you find anything repetitive or confusing; g) does any question need more explanation; h) how did you find the questions; i) any further comments. The questionnaire was revised based on the participants’ suggestions to draw the final survey and the schedule.

The closed-ended quantitative survey questionnaire obtained a numeric description of the sampled population’s perception [26]. There were 39 items with a five-point level of agreement Likert scale, among which 11 items were used to capture the perception regarding the risk factors (causes), 23 items were used to capture the intention and actions towards weight management, and 5 items were used to capture the coping measures to manage their body weight, whose Cronbach’s alpha was 0.81. Further, there were another 6 items with a two-point dichotomous Likert scale, among which 4 items were used to capture the participant’s perception of obesity and 2 items captured the participants’ intention towards their body weight, whose Cronbach’s alpha was 0.49. We made the quantitative survey questions available in English, and Bengali, the regional language, which three local Bengali speakers translated. It took around 10 minutes to finish the survey.

In alignment with the research problem and quantitative survey questionnaire, we drew a semi-structured interview guide containing open-ended questions to better understand the target population’s perception. Face-to-face interviews were conducted, and the discussions were audio-recorded. In total, there were fourteen semi-structured questions. Of the fourteen qualitative questions, two specifically inquired about the perception of excess weight; eleven specifically inquired about intention and actions towards weight management and preventive strategies and one more question was asked to begin the conversation. The interviews were administered among voluntarily agreed-upon participants in their everyday language (Bengali, English, and Hindi) so that the interviewees could freely express their opinions. It took an average of 20 to 25 minutes to finish an interview.

### Field experiences

The principal investigator visited the field between November 2019 and October 2020. Conducting questionnaires and interviews was difficult and inconvenient because of weight-related stigma, lack of interest and motivation, time constraints, and the ongoing covid-19 pandemic during the study period. The prevalence of excess weight (BMI > = 25) in Kolkata was 40.83%, according to the NFHS-4, 2015–16. Given a 10% of margin of error, 40.83% prevalence rate with z score = 1.96, the minimum Sample Size for Kolkata was 93 (= (1.96)^2^ * 0.4083*(1–0.4083) / (0.10)^2^). However, we extended the sample size up to 120 for quantitative questionnaires. In addition, we conducted 18 in-depth interviews.

### Analysis of data

#### Univariate analysis

Using Stata 14.0, we calculated descriptive statistics to show the percentages and frequency of the demographics and responses of the sampled participants. By entering each participant’s weight (kg) and height (cm) into the National Institutes of Health (NIH) website, we estimated the BMI.

#### Coding of themes

We opted for inductive coding. Creswell [26] outlined six steps for analysing qualitative data, which we followed: a) Writing up the field notes and transcribing the audio recording, b) Re-reading the data to reflect on the general meaning and credibility of the transcripts, c) Creating a code for the appropriate parts that is an abbreviation of the given topic, d) Additionally, codes are created to establish broad themes as a representation of the study findings, e) relating the narratives to the themes, f) interpreting the qualitative data in light of the literature. The lead investigator transcribed the interviews, a time-consuming and labour-intensive process requiring about 1–2 hours per interview. We focused on five major themes in this research.

#### Merging results of two datasets

We merged, compared and presented the qualitative and quantitative results. We also used ‘joint displays’ by juxtaposing (side-by-side) the qualitative and quantitative findings [29].

## Results

### Descriptive statistics of the survey participants

A total of 120 people participated in the study, 60 of whom were females and 60 of whom were males. Table 1 shows the descriptive statistics of study participants aged 25 to 54, with an average age of 39. The sampled participants were more present between the ages of 25 and 34. Half of the respondents had obtained their diplomas or graduated. Most participants had a monthly family income between 40,001 and 80,000 INR, mostly employed, with a household size of around four persons. Most survey participants believed in Hinduism, belonged to the General or OBC social group, and spoke Bengali as their first language. The majority of the participants were married with children. Further, more participants had a BMI greater than 30, a waist circumference greater than 102 cm, and were nonsmokers. The sampled participants had a mean BMI of 31.28 and a waist circumference of 106.44 cm. Also, 45.83% of the survey participants never drank alcohol, and one-fifth had thyroid problems.

**Table 1:** Descriptive statistics of the survey participants, Kolkata, 2019–20

Background Characteristics	Frequency (n)	Percentage (%)
**Age (in years)**		
25–34	44	36.67
35–44	33	27.5
45–54	43	35.83
**Education Level**		
Up to Secondary	15	12.5
Up to Graduation	65	54.17
P.G. or Higher	40	33.33
**Monthly Family Income**		
Up to 40,000	25	20.83
40,001 to 80,000	39	32.5
Above 80,000	37	30.83
Don’t Know/Prefer not to answer	19	15.83
**Employment Status**		
Not employed (Unpaid)	27	22.50
Employed	93	77.50
**Household Members**		
Up to five members	112	93.33
More than five members	8	6.67
**Religious Beliefs**		
Hinduism	85	70.83
Islam	5	4.17
Other	30	25
**Social Group**		
SC/ST	8	6.67
General/OBC	105	87.5
Can’t Say/Don’t Know	7	5.83
**Mother Tongue**		
Bengali	109	90.83
Others	11	9.17
**Marital Status**		
Unmarried	31	25.83
Married	89	74.17
**Have Children**		
Yes	79	65.83
No	41	34.17
**Body Mass Index**		
25 or higher	59	49.17
30 or higher	61	50.83
**Waist Circumference**		
Up to 102 cm	51	42.5
More than 102 cm	69	57.5
**Smoking**		
Regular	31	25.83
Occasional	14	11.67
Former	8	6.67
Never	67	55.83
**Alcohol Consumption**		
Regular	10	8.33
Occasional	53	44.17
Former	2	1.67
Never	55	45.83
**Health Problems**^a^		
PCOS^b^	8	13.33
Thyroid	25	20.83
Arthritis	6	5.00
Acidity/Gas	4	3.33
Mental health condition	4	3.33
Hypertension	45	37.50
High blood sugar	20	16.67
Sleep apnea	26	21.67
Asthma	13	10.83
Cholesterol	24	20
Other health issues	12	10
No health problem	35	29.17
**Total**	120	

Source: Primary Survey, Kolkata, 2019-20

^a^Had suffered or presently living with the specific health problems and/or under the medication

^b^Among 60 female participants

### Descriptive statistics of the in-depth interviewees

A total of 18 people, nine males and nine females, volunteered to participate in the interview. (Table 2). The average age of the interviewees was 39 years, and they were between 27 to 52 years. Most participants were graduates and employed, with a monthly family income of more than 80,000 INR, and lived in households with an average of four people. Hinduism was the most prevalent religious belief among the sampled interviewees, who primarily belonged to the ‘General’ social category and spoke Bengali. The majority of those interviewed were married with children. In addition, most of the interviewees in the sample had a BMI of more than 30 and a waist circumference of more than 102 cm. The sampled participants had a mean BMI of 32.87 and a waist circumference of 109.28 cm. Furthermore, nearly half of the participants said they had never smoked, only drank alcohol occasionally, had a minimum of one health problem or were on medication.

**Table 2:** Descriptive statistics of the interviewees, Kolkata, 2019-20

Partcicipant No.	Age (in years)	Sex	Level of education	Occupation	Monthly family income (INR)	Household members	Religious belief	Caste	Mother tongue	Marital status	Have Children	BMI	WC (in cm)	Smoking	Alcohol consumption	Known complication/ under medication
1	27	Female	Post Graduate	Service	Above 80,000	4	Hinduism	General	Bengali	Unmarried	No	33.5	117.5	Never	Occasional	N/A
2	31	Female	Post Graduate	Service	Above 80,000	4	Hinduism	General	Bengali	Married	Yes	30.3	105	Occasional	Occasional	N/A
3	33	Female	Graduate	Business	Above 80,000	4	Hinduism	General	Bengali	Married	No	37.3	123	Never	Former	PCOS; Hypertension; Cholesterol
4	37	Female	Graduate	Business	Above 80,000	3	Hinduism	OBC	Hindi/Bhojpuri	Married	Yes	26.6	98	Never	Occasional	Thyroid
5	38	Female	9th Standard	Homemaker	Don’t Know	4	Hinduism	Can’t Say/Don’t Know	Bengali	Married	Yes	26.5	93	Never	Never	N/A
6	40	Female	Post Graduate	Service	40,001–80,000	4	Omnism	General	Bengali	Separated	Yes	35.9	102	Never	Never	N/A
7	45	Female	Graduate	Homemaker	Above 80,000	3	Hinduism	General	Bengali	Married	Yes	36.3	112	Never	Never	PCOS; Thyroid; Type 2 Diabetes; Hypertension
8	46	Female	Graduate	Freelance Writer	Up to 40,000	3	Hinduism	General	Bengali	Married	Yes	31.6	99.5	Occasional	Occasional	Migraine
9	51	Female	Graduate	Freelance Researcher	Above 80,000	4	Islam	No Caste	Bengali	Married	Yes	41.5	119	Occasional	Occasional	Type 2 Diabetes; Hypertension
10	26	Male	Post Graduate	Private Tutor	40,001–80,000	4	Humanism	ST	Bengali	Unmarried	No	33.3	111	Occasional	Occasional	N/A
11	28	Male	Post Graduate	Research Scholar	Above 80,000	4	Hinduism	General	Bengali	Unmarried	No	33.2	108	Occasional	Occasional	N/A
12	30	Male	Post Graduate	Private Tutor	40,001–80,000	3	Non-believer	General	Bengali	Unmarried	No	27.1	96	Occasional	Occasional	Asthma
13	37	Male	Higher Secondary	Service	Up to 40,000	2	Non-believer	General	Bengali	Unmarried	No	31.6	104	Never	Occasional	Gas
14	41	Male	Graduate	Business	40,001–80,000	5	Hinduism	General	Bengali	Married	Yes	31.4	111	Regular	Occasional	Type 2 Diabetes; Hypertension
15	43	Male	Graduate	Service & Business	Up to 40,000	8	Hinduism	General	Bengali	Married	Yes	34.3	121	Never	Never	High Blood Sugar
16	47	Male	Graduate	Service	40,001–80,000	2	Hinduism	General	Bengali	Widowed	Yes	30.8	106	Regular	Former	N/A
17	50	Male	Higher Secondary	Business	40,001–80,000	4	Humanism	General	Bengali	Married	Yes	37.5	127	Never	Regular	Hypertension; Cholesterol
18	52	Male	Graduate	Service/Author	Above 80,000	2	Non-believer	General	Bengali	Married	Yes	32.9	114	Former	Occasional	Type 2 Diabetes; Hypertension

Source: Primary Survey, Kolkata, 2019-20

### Themes

Five broad themes based on participants’ perception, knowledge and health behaviours emerged from the qualitative data: 1) risk factors; 2) benefits of healthy behaviours; 3) physical activity behaviour; 4) dietary behaviour; 5) obesity prevention.

### Risk factors

#### Obesity

Survey participants felt that obesity was a health condition (82.91%), disease (61.67%), the result of lifestyle change (85%) and not a lifestyle choice (68.64%).

Interview participants confirmed lifestyle as one of the most important reasons for obesity. The view of having excess weight as unhealthy was high among young adults and females. Participant 8 claimed, *“At this time, the influence in your generation and the later generation, for them lifestyle is the culprit, which is deadly.”*

Conversely, Participant 3 shared her views on obesity and opined, *“It is like a disease, something we are unable to fix. I think obesity is a concern more than a disease. It is not a disease. It is like sitting on a time bomb.”*

Differently, Participant 14 stated, *“Obesity, to an extent, is an illness as it affects both physical and mental health.”*

An interviewee emphasised the beauty industry’s impact on how obesity is perceived, further expanding the understanding. He shared, *“I don’t have a medical understanding of obesity. What my personal understanding is that the cosmetic industry, the styling industry and the fashion industry have brought in a lot of perceptions of how you would look to others. And one of the key factors here is obesity. So I feel there are two kinds of obesity – one is obesity which is normal and healthy. You could still be healthy and be obese, and other is when you are not normal and healthy, and you are obese. That needs medical intervention.”*

#### Family

As per the survey respondents, family’s eating habits (56.67% agree; 27.50% strongly agree) and family history of obesity (60% agree; 13.33% strongly agree) influenced the risk of obesity.

Participant 3 confirmed, *“Family plays an important role. Only if we were told to eat fruits every day. We don’t eat fruits. A healthy diet should consist of fruits, leafy vegetables, and fibre rich. These things were not imbibed. So those food habits stayed on. When hungry, often, I go for instant noodles.”*

On family history, participant 6 confirmed, *“I have never seen my parents slim and trim. Most of my family members - they are apparently not the healthy perfect that we understand. We should be. It is more than slightly what is required we have always carried. It could be genetic reasons.”*

#### Behavioural factors

Two of the most critical risk factors of obesity were eating foods with too much fat and sugar (42.50% agree; 54.17% strongly agree) and lack of physical activity (40.83% agree; 53.33% strongly agree). The survey participants also revealed a lack of sleep (42.50% agree; 19.17% strongly agree) as the other risk factor.

According to the interview participants, a lack of physical activity is another significant cause of obesity. Participant 10 expanded, *“People are not engaged in any physical activity. After eating, what people should do regarding physical activity but are not doing or are unable to do so. People are availing Ola or Uber. Rapido is new now, and many people are availing that for transportation. Therefore, what is happening is that the calorie that they should burn with some physical activity, but hardly people are engaging in physical activity.”*

#### Sedentary activities [TV, computer, mobile, etc.] and stress

As per the survey respondents, hours of TV watching, phone browsing, indoor gaming (44.17% agree; 22.50% strongly agree) and constant stress (40% agree; 22.50% strongly agree) influenced the risk of obesity.

Participant 4 confirmed and summarised a few important risk factors of excess weight, *“Our lifestyle – previously we used to walk for hours, spend time at playgrounds but now our life has become restricted. TV, mobile, gadgets, all these affect our health too much. Obesity is, therefore, caused due to junk food and lifestyle. People in a job sitting on a chair for the entire day. People who are at home watch TV or browse the phone. Due to the building, there is less space. Stress is also the most important thing for obesity. And also not eating timely.”*

#### Time-poor

Lack of time and work pressure were risk factors since participants were discouraged from engaging in physical activity (48.33% agree; 20.83% strongly agree).

Participant 14, who was already diagnosed with Type 2 diabetes and hypertension before the age of 41 and ran his own business, confirmed and explained, *“For surviving, the earning you need, to earn that money, the middle class are the worst sufferer. Maximum problems belong to the middle class. The lower and upper classes have fewer fat people, comparatively, and rich people can manage their working hours accordingly. They can manage their time to go to the gym for an hour. For me, that is not possible, leaving my shop unattended. If I go to the gym, I have to keep a worker to whom I got to pay a certain amount of money.”*

#### Wealth

Survey participants mostly disagreed that excess weight is a sign of prosperity (27.50% disagree; 27.50% strongly disagree) influencing the risk; and that the lack of money increased obesity risk (42.50% disagree; 35.83% strongly disagree).

Interview participant 10 expanded, *“So there is an entirely low-income group who may have had sources to different kinds of food, may not have access to those foods anymore. Obesity is for all classes. But if we do an analysis, particularly in the slum areas, there is an entire baby who is obese in that sense. People could be undernourished and obese at the same time.”*

#### Urbanisation

Urbanisation in Kolkata (41.67% agree, 13.33% strongly agree) influenced the risk of obesity.

Participant 15 confirmed, *“Young generations are also taking escalators now. Instead, if they take stairs, that can also be an exercise. Small changes can bring a bigger impact. It is becoming difficult after getting extra benefits. In villages transport facility is poor, so people walk. They have to take the stairs. But in the urban city, getting extra advantages is becoming problematic, I think. And these junk fried, oily foods are not there in villages.”*

Furthermore, qualitative interviews expanded the understanding that technology, digital marketing of food, lack of knowledge and self-care influenced the risk of obesity. During the field visit, the PI observed that generally, people lived a busy life and were reliant on technology and app-based marketing, takeaway and readymade foods were popular, and people lacked awareness and health care. For example, participant 13 stated, *“Yes, now our country has developed a lot, therefore, swiggy, uber eats, all kinds of home delivery and free services. So I think with more passing days, the consumption of these free services are affecting health. Our heads do not think twice about what these free services do to our bodies. People are very excited to order, but we don’t understand how unhealthy that is. That’s the problem.”*

### Benefits of healthy behaviours

Table 3 compares quantitative and qualitative findings on the benefits of healthy behaviours in a joint display. The first column shows the qualitative results, the second column shows the quantitative findings, and the last column suggests the fit of data integration, whether the findings are confirming, discording or expanding. When we use the terms “concordance” and “expansion,” we mean that the two data forms agree, suggesting the same thing and that the knowledge of the subject is expanding, respectively.

**Table 3:** Juxtaposed findings of quantitative and qualitative investigation on the benefits of healthy behaviours

Qualitative interview findings	Quantitative survey findings	Type of integration
Participants responded that they intended to lose weight. Participant 6 voiced, *“I intend to reduce weight. I fear knee pain and all. I don’t have so much money to repair the damages in my body.” *Contrarily, Participant 8 said, *“If it happens, then good, but not such intention. Maybe manage a little bit.”*	96.67% of the respondents wanted to lose weight, and only 3.33% wanted to stay the same weight.	Concordance
Several individuals urged to become fit, healthy, and energetic. Furthermore, Participant 18 reported that after making changes to his diet, he began to feel better. *“I am doing it because last month, when I was getting up in the morning, I was not feeling too well. Then I felt I needed to do something drastic to my diet. Because I was eating more than I should be eating. And I was eating a lot of nonsense stuff.”*	The respondent felt they wanted to lose weight because they wanted to feel healthy (50.83% agree, 45.83% strongly agree).	Concordance
Physical activity and a healthy diet were frequently suggested as ways to maintain good physical and mental health as well as to maintain a healthy weight. Participant 14 opined, *“Of course, your health will feel good. Your mental health, your heart will feel good. There is a relation between physical and mental health.”* Likewise, few stated that physical activity improved blood circulation and kept the body active, whilst healthy food improved digestion. Participant 12 said, *“Combination of healthy eating with exercise – you will be physically fit. You can avoid different types of disease which are very prevalent.”*	97.50%, and 87.40% agreed that they wanted to maintain a healthy weight to be fit, have more energy (45.83% agree, 51.67% strongly agree), and manage their stress and anxiety (47.90% agree, 39.50% strongly agree).	Expansion

### Physical activity behaviours

Table 4 shows a side-by-side display of quantitative and qualitative findings on physical activity. The green colours denote agreement, the blue colours denote disagreement, and the grey ones denote inconclusive survey participants’ responses.

**Table 4:**
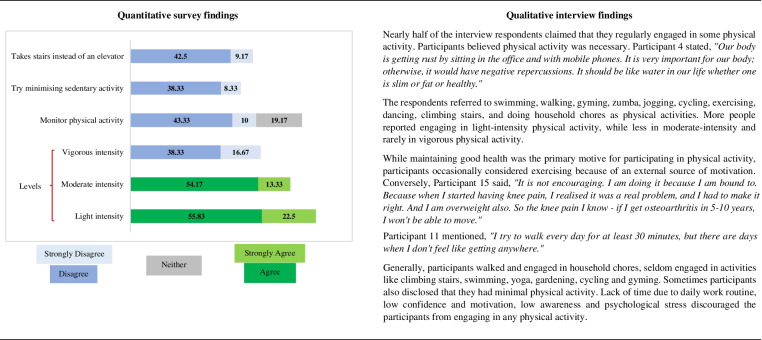
Juxtaposed findings of qu﻿antitative and qualitative investigation on physical activity be﻿haviours

### Dietary behaviour

#### Calorie consumption

Survey participants frequently disagreed about being aware of their calorie consumption (45.83% disagree & 15% strongly disagree) and did not track their food or liquid intake (45% disagree & 16.67% strongly disagree).

Interview participants confirmed that they seldom had an idea of their calorie consumption. Participant 3 expanded, “*When I referred a nutritionist, they said I should consume 1500-1700 calories and eventually lessen it. Then I went till 1300. Now at least I consume 2500 calories per day. So no calorie deficit is there*.”

#### Read nutritional labels

More than half of the survey participants agreed to read nutritional labels (43.33% agree or strongly agree). However, 39.17% of the participants disagreed with not consulting dietary labels.

Conversely, interviewees recalled that they did not consult dietary labels.

#### Consumption of high-calorie food

Generally, survey participants agreed that they limited their consumption of high-calorie food (50% agree; 11.67% strongly agree) and limited their eating portions (58.33% agree/strongly agree).

In discordance, interview participants recalled eating high-calorie and low-nutrient-dense foods.

“After that in the evening, I crave for something some bad stuff – snacks” – Participant 10.

Occasionally interviewees stated that they ate small portions. For example, participant 7 stated, “*Intention towards my weight is eating less. Not to eat much. I intend to lose weight. Only one intention. If that makes me sad, that is okay. One or two days I can eat good food, most days bad food*.”

#### Dietary pattern and food knowledge

More survey participants agreed that they consumed low-fat meals (52.50% agree; 13.33% strongly agree), low-carbohydrate meals (55% agree/ strongly agree) and high-fibre foods (56.67% agree/ strongly agree); monitored their consumption of butter, cream and cheese (47.50% agree; 12.50% strongly agree); opted drinking water over sugary drinks (46.67% agree & 16.67% strongly agree) to prevent obesity.

However, the dietary recalls of the interview participants were contrary. For example, interview participants skipped their daily intake of fruits and vegetables.

“My biggest drawback is I do not eat vegetables and fruits because of that the way to start eating it is very difficult.” - Participant 8.

The PI observed that individuals tended to understand food and nutrition poorly. For an, e.g., one survey participant mentioned that she did not eat fruits so to avoid sugar intake.

Additionally, the qualitative interview provided more detail and shed light on the lack of dietary knowledge, high sugar and low protein consumption. Seldom interview participants were aware of low-fat, low-carbohydrate meals and high-fibre foods. As per those participants, vegetables were low-fat foods; less rice and roti, protein-rich diet were considered low carbohydrate meals; while high-fibre foods were mainly multi-grains. On the other hand, Participant 9 said with the sentiment, “*I really don’t know because previously, we used to hear not to eat eggs. Now the new fad is ‘keto’. Now keto is based on eggs, good fats. Previously there was a saying not to eat ghee. Now it’s a superfood. Butter was not suggested; now it’s another kind of a superfood. ‘Cholesterol is bad’, now I hear cholesterol is good for you. You should not be lowering cholesterol below your level because it has its benefits to break down this and that. Previously it was the HDL is good, LDL is bad. Now I don’t hear that as well. Now they are saying if your LDL decrease to a low level, it creates a problem with your digestion. So I don’t know*.”

Participant 4 felt people were the “culprit” for their health. She added, “*I like noodles. It’s my favourite, but when I conceived, I was asked to quit as it is made of refined wheat, and it takes four days to digest. These are wax coated. We do not know all these. But it is totally unhealthy. Only taste!*”

While Participant 14 reasoned, “*Our understanding is all made up of what is healthy food. Some are made up by doctors and some by media advertisement*.”

#### Frequency of eating outside

Most survey participants agreed that they avoided eating outside and preferred eating at home (42.50% agree; 19.17% strongly agree) to prevent obesity.

In contrast, interviewees repeatedly recalled eating at least 4–5 days a month outside their homes.

“Typically I eat one meal at home and rest outside” – Participant 3.

The PI observed that outside eateries and food deliveries were highly prevalent, roadside shops were filled with ready-made instant food items, and people tended to meet over food. The qualitative interviews contribute to a better understanding of how the food environment has changed, possibly due to the convenience of ready-made food.

“The foodscape has changed in different ways, and the restaurant industry has boomed across the city. So for a long time, I remember going to the restaurants was a kind of most discussed affair in the family. Now it is not a matter; you can go and have everyday food.” – Participant 10.

“It is because of ease. As people are cooking less now that restaurants and food chains have increased a lot. Within the last 21 years, there has been a huge difference.” – (Participant 2).

Similarly, qualitative interviews expanded the understanding that socialisation took place around food. Participant 10 referred to a fascinating insight related to food and diet, “*my obesity is not simply about me. It’s the food culture that we have adapted so easily. We are made to imbibe through socialisation. Nowadays a typical socialising point will be a fast food centre or a restaurant which I remember not to be. Rather it used to be a friend’s place earlier, or it could be a playground for a long time because we could not afford to get priced expensive food. But now the typical socialising point would be KFC, Mc Donald’s. There is so much promotion around most of these foods rather than any organic food market. Only burgers and all. Even what we call healthy food also to be contested*.”

### Obesity prevention

Table 5 shows another side-by-side display of quantitative and qualitative findings on obesity prevention. Green colours indicate agreement, and blue colours indicate disagreement.

**Table 5:**
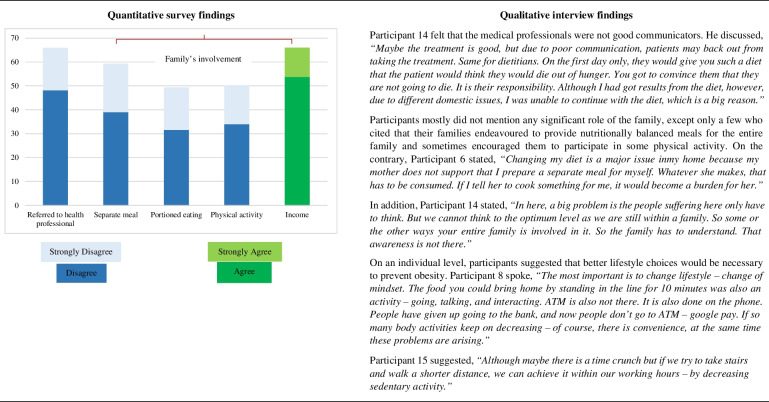
Juxtaposed findings of quantitative and qualitative investigation on obesity prevention

## Discussion

A few survey and occasionally the interview participants indicated being unfamiliar with the term “obesity”; however, they were familiar with the word ‘fat’. According to most survey and interview responses, obesity results from lifestyle changes.

Interview participants explained that the current lifestyle is characterised by sedentary jobs with long working hours, excessive use of motorised vehicles, a life restricted to TV, mobile phones and computers, poor food habits, depression, and substance abuse. The results further suggested that participants had the basic understanding that family history of obesity and eating habits, lack of physical activity [6], fatty and sugary foods, stress, and sedentary activities increased the risk of obesity. Wealth played an interesting role that seemed to influence the risk of obesity among people of different economic statuses. Findings also revealed a lack of time to participate in physical activity and urbanisation as the other risk factors of obesity. Interview findings added a few additional factors, such as technology, lack of health knowledge and self-care, that influenced the obesity risk.

Based on the literature, television viewing may increase the risk of obesity by influencing the overall levels of energy expenditure [30]. In similar ways, mobile and computer browsing can also encourage sedentary activity and influence the risk of obesity, as has been implied in the present study. Stress has long been associated with weight gain [12, 31, 32], comparable to how participants perceive it as a risk factor. Consistent with the results of this study, prior studies have evidenced that time poverty could be an obstacle to exercising and eating for people with long, sedentary and inflexible working hours [15]. At the same time, obesity rates are likely to be higher in urban localities [33, 34].

Technological advancements such as vehicle apps, food delivery apps that provide discounts, and a slew of other marketing apps may have made life easier on the one hand; nonetheless, they’ve also caused people to walk less and eat more on the other. Furthermore, as there is more use of cars and less cycling and walking, it results in much lesser physical activity, which contributes to the rising obesity rate, as also has been evidenced in the literature [22]. This is how technology positively influences the obesity rate and is consistent with prior studies.

The perceived susceptibility of the participants influenced their weight-loss intention the most. Consistent with the results from previous studies, interview participants intended to make lifestyle changes by switching to a healthier diet and increasing their physical activity [4] or engaging only in physical activity [20] for their well-being. Nevertheless, interview participants felt the importance and were aware of the several benefits of healthy eating and physical activity [5] that it helped with physical and mental well-being.

In this study, interview participants were asked to recall their physical activities on regular days, while survey participants were questioned on the intensity of physical activity against the level of agreement. Interview participants majorly believed in the necessity of physical activity and were aware of various physical activities. However, the physical activity level appeared to be less than the recommended level. Only 35% of the survey participants choosing to take the stairs instead of an elevator merely suggests a low level of activeness among the participants. 12.5% (*n* = 15) and 38.89% of the survey and interview participants did not engage in any intensity of physical activities. Besides, the survey participants did not monitor their physical activity levels, while studies suggested that monitoring activity levels could address energy imbalance [9] and could help with weight management [13]. The majority of interview participants felt encouraged to participate in physical activity, primarily to improve their health, and when there was a perceived severity or susceptibility or an external cue such as the desired outfit, inspiration from others’ healthy behavioural habits, or someone else’s weight loss success. On the contrary, occasionally, participants felt discouraged from participating in physical activity due to lack of time and work pressure, lack of interest, poor self-efficacy, low motivation, lack of awareness, and poor mental health.

As it appears, in an urban city like Kolkata, a shift in the occupational job structure being more sedentary is resulting in an overall drop in energy expenditure. As found in the current study, widespread mechanisation of labour [30], automated transportation, excessive use of technology, and a lack of physical exercise in daily routines [22] had further decreased the amount of energy expenditure. On the other hand, it was inferred that other activities demanding time, such as longer working hours or domestic responsibilities, made it challenging to engage in physical activities, as was also found in a previous study [30]. Furthermore, studies suggested that poor mental health might reduce one’s interest in engaging in physical activity while increasing the probability of stress eating [4], as mentioned by two interview participants in this study.

A discordance of findings on dietary behaviours between the survey and interview participants was observed. A significant proportion of the survey participants agreed to have consumed low-carbohydrate and low-fat meals. In contrast, the interview participants’ dietary recall showed different findings. Three interview participants narrated following a low carbohydrate diet or a nutritionist-suggested diet; however, they could not continue it and regained their weight. Although a reasonable proportion of survey participants agreed that they consumed high-fibre foods to prevent obesity, interview participants informed skipping their daily intake of fruits and sometimes vegetables, which are known to be the primary source of dietary fibre for the human body. Yet, the interviewees believed these were an integral part of a healthy balanced diet. Survey participants mostly agreed that they avoided eating outside while preferred eating at home, read nutritional labels, and had limited consumption of high-calorie food. On the other hand, interviewees repeatedly recalled eating at least 4–5 days a month outside their homes, not consulting dietary labels, and eating high-calorie and low-nutrient-dense foods. This divergence in findings could be because the survey participants did not understand the question or had poor educational awareness and misinformation regarding food and nutrition.

Consistent with Bray’s [35] weight cycling, our study also witnessed individuals who lost weight through dieting, then stopped, regained their weight, and sometimes even more. Further, the spreading of misinformation is usually intended to make a profit [36]. It is plausible that these medical and pharmaceutical industries are in the business of treating sick people rather than preventing them [37] with a lack of intent to adopt diet therapy [36]. That has also been evidenced in this study, as suggested by interviewees. The food environment has been changing over the last few decades [11], as has been witnessed in this study. There has been a considerable increase in the heavily promoted, readily available processed foods that are low in nutrients and high in calories, consistent with a previous study [11]. A few of the most common sources of market-shelved processed foods in the interview participants’ diet were biscuits, sweets, Maggi, noodles, etc. These low-nutrient foods are designed to smell and look better to attract customers [38], whereas nutrient-dense foods, on the other hand, are frequently labelled as “bad food,” as this study demonstrates.

In the present study, sugar consumption through foods appeared to be high, whereas protein consumption appeared low as per the interview participants’ dietary recall. Added sugar makes people addicted to those foods without their knowledge [39], and there can be as many as 70 different names of sugar on the food packages [36] as a way to hide it [38]. The so-called 500 g health drinks packs have at least 16 teaspoons of added sugar, the so-called 500 g health biscuits have a minimum of 17 teaspoons of added sugar, while 500 ml of a cola drink has around 14 teaspoons of added sugar. These higher energy densities of certain foods could also be another driver behind the obesity epidemic. Further, fewer participants were aware of their calorie count or tracked their food and liquid consumption. Interview participants indicated that nowadays, socialisation revolves around food [10] because that is the simplest way people tend to communicate and celebrate [40] in present days, and in general, these are palatable fast foods, as was also found in earlier studies. Finally, interviewees has implied that there is ample food advertisement, majorly for westernised fast food; however, there is no promotion or discussion on healthy food and  right nutrition. Hence, these need to be challenged.

At an individual level, few survey respondents had sought the advice of a health professional or a dietitian, and occasionally interview participants hinted that they lacked good communication skills. At an interpersonal level, many survey participants and their families had the income to prevent obesity. Aside from that, the family’s participation was implied to be trivial. Occasionally interviewed participants indicated that the family was a risk factor due to a discouraging and unsupported family environment, as was also evidenced in a previous study [41]. That was because of a poor understanding of nutrition and health. As per the interview participants and an earlier research work [9], better lifestyle choices, such as choosing healthier whole foods, increasing physical activity, and minimising sedentary activities, is needed at an individual level to prevent obesity.

## Conclusions

The present study assessed the perceptions, risk factors and health behaviours among adults with excess weight in the Kolkata metropolitan area, India using a concurrent mixed methods approach. Our sampled population comprised people from different educational, religious, social and economic groups. The study documented the association of low awareness, lifestyle and behavioural factors, family history, stress, time-poor, sociocultural factors, and urbanisation with excess body weight. Firstly, educating and promoting healthy eating habits and physical activity is essential. It is necessary to inform residents about lifestyle changes that occur due to urbanisation, such as the diet, the impact of excessive use of technology on physical activity levels, and how these changes affect their lives. Increasing whole foods in the diet such as more vegetables, fruits, nuts, etc., substituting with healthier available options such as swapping juices with whole fruits, minimising sedentary activity, such as taking the stairs instead of the elevator, cycling or walking for shorter distances instead of taking mechanised vehicles, monitoring intake and level of activity can all help to maintain and improve good health and also be sustainable. Promoting a participatory culture and behavioural changes at the community level may prove helpful. Secondly, the findings revealed a lack of social support. Hence, extending social support may help reduce feelings of isolation. Further, healthy lifestyle choices in the family can influence the family’s food and activity pattern. The setting of healthy behaviours from the children can help reduce childhood obesity and can assist in bringing down overall obesity prevalence. Thirdly, since many participants reported a lack of time and work pressure, better policies are needed so that people can make time and prioritise their health equitably. Lastly, healthcare services need to improve, and patients with obesity should be treated with more compassion since it is a complex chronic disease and there is an absence of specialist obesity treatment in India [42]. Instead of just treating diseases and other complications, health practitioners need to empower their clients with health education in order for them to postpone or prevent them.

There are a few shortcomings in this research. Finding participants was the most difficult aspect of this study. Participants had to be contacted with sensitivity since there was a chance of weight-related stigma. Lack of enthusiasm, time and a lack of incentive and stigma were possible causes for the low participation rate. This study’s use of purposive and snowball sampling procedures may have resulted in selection bias. Although, the PI made every effort to gather as many participants as possible from around the city. Furthermore, during the covid-19 outbreak and the country’s lockdown, conducting interviews and surveys had become more challenging, resulting in a low response rate. The majority of survey items were subjective, which could have led to confounding. Finally, since the study is based in the metropolitan urban setting, the responses may vary in other geographical settings.

Yet, this study has numerous merits. This study kept an equal representation of female & male volunteers and attempted to address the problem holistically using a mixed methods approach. The PI compiled two forms of data that aided in understanding the complexity of the problem and took measurements of each participant’s height, weight, and waist circumference. This research should be able to contribute to the design of future studies and future courses of action for necessary interventions.

### Regarding dual publication

Figure 1 ‘Diagram of Concurrent Mixed Methods Design’ in the methods section, Table 1 ‘Descriptive Statistics of the Survey Participants’ & Table 2 ‘Descriptive Statistics of the Interviewees’ in the results section can be the same as one of our other manuscripts. Since this study is based on a primary survey employing a concurrent mixed methods design, the number of survey respondents and identified interviewees are the same. None of the other main results matches any manuscripts that would be published or under consideration. Hence, this does not constitute a dual publication.

## Supplementary Information

**Additional file 1: Table 1.** Descriptive statistics of survey items.

## Data Availability

The datasets generated and/or analysed during the current study are not publicly available since this study is based on a primary survey but are available from the corresponding author on reasonable request. A supplementary appendix file has been provided for the survey items.

## Abbreviations

BMI: Body Mass Index
HDL: High-density lipoprotein
IERB: Institutional Ethics Review Board
IIPS: International Institute for Population Science
JNU: Jawaharlal Nehru University
LDL: Low-density lipoprotein
NFHS: National Family Health Survey
OBC: Other Backward Classes
PCOS: Polycystic ovary syndrome
P.G: Post Graduation
PI: Principal Investigator
WC: Waist circumference
WHO: World Health Organization
WOF: World Obesity Federation
