# Supplementary material for: Perception, risk factors, and health behaviours in adult obesity in Kolkata, India: a mixed methods approach
Source: BMC Public Health. 2022 Dec 19;22:2376. doi: 10.1186/s12889-022-14531-9 (PMC9761027; doi:10.1186/s12889-022-14531-9)
Supplement: Supplementary file 1 — Additional file 1: Table 1. Descriptive statistics of survey items. [file 12889_2022_14531_MOESM1_ESM.docx]

| **Appendix Table 1. Descriptive statistics of survey items** | | | |  |  |  |  |
| --- | --- | --- | --- | --- | --- | --- | --- |
| **Serial No.** | **Survey Items** | **Responses** | | | | | **Missing** |
|  |  | **Yes** | **No** |  |  |  |  |
| 1 | Health condition | 97 (82.91%) | 20 (17.09%) |  |  |  | 3 |
| 2 | Disease | 74 (61.67%) | 46 (38.33%) |  |  |  |  |
| 3 | Lifestyle choice | 37 (31.36%) | 81 (68.64%) |  |  |  | 2 |
| 4 | Consequence of lifestyle change | 102 (85%) | 18 (15%) |  |  |  |  |
|  |  | **Strongly disagree** | **Disagree** | **Neither** | **Agree** | **Strongly agree** |  |
| 5 | Family history of obesity | 4 (3.33%) | 16 (13.33%) | 12 (10%) | 72 (60%) | 16 (13.33%) |  |
| 6 | Family's eating habits | 3 (2.5%) | 10 (8.33%) | 6 (5%) | 68 (56.67%) | 33 (27.5%) |  |
| 7 | Lack of sleep | 9 (7.5%) | 24 (20%) | 13 (10.83%) | 51 (42.5%) | 23 (19.17%) |  |
| 8 | Lack of physical activity | 3 (2.5%) | 4 (3.33%) |  | 49 (40.83%) | 64 (53.33%) |  |
| 9 | Constant stress | 9 (7.5%) | 19 (15.58%) | 17 (14.17%) | 48 (40%) | 27 (22.5%) |  |
| 10 | Fatty and sugary foods | 2 (1.67%) | 2 (1.67%) |  | 51 (42.5%) | 65 (54.17%) |  |
| 11 | Watching long hours of television, browsing mobile phone, playing indoor games | 7 (5.83%) | 15 (12.5%) | 18 (15%) | 53 (44.17%) | 27 (22.5%) |  |
| 12 | Sign of prosperity | 33 (27.5%) | 33 (27.5%) | 12 (10%) | 29 (24.19%) | 13 (10.83%) |  |
| 13 | Lack of money | 43 (35.83%) | 51 (42.5%) | 12 (10%) | 9 (7.5%) | 5 (4.17%) |  |
| 14 | Time and work | 9 (7.5%) | 20 (16.67%) | 8 (6.67%) | 58 (48.33%) | 25 (20.83%) |  |
| 15 | Urbanisation | 10 (8.33%) | 22 (18.33%) | 22 (18.33%) | 50 (41.67%) | 16 (13.33%) |  |
|  |  | **Yes** | **No** |  |  |  |  |
| 16 | Same weight | 4 (3.33%) | 116 (96.67%) |  |  |  |  |
| 17 | Lose weight | 116 (96.67%) | 4 (3.33%) |  |  |  |  |
|  |  | **Strongly disagree** | **Disagree** | **Neither** | **Agree** | **Strongly agree** |  |
| 18 | Fit and have more energy | 1 (0.83%) | 1 (0.83%) | 1 (0.83%) | 55 (45.83%) | 62 (51.67%) |  |
| 19 | Stress and anxiety | 1 (0.84%) | 5 (4.2%) | 9 (7.56%) | 57 (47.9%) | 47 (39.5%) | 1 |
| 20 | Feel healthy | 1 (0.83%) |  | 3 (2.5%) | 61 (50.83%) | 55 (45.83%) |  |
| 21 | Light intensity | 4 (3.33%) | 9 (7.5%) | 13 (10.83%) | 67 (55.83%) | 27 (22.5%) |  |
| 22 | Moderate-intensity | 5 (4.17%) | 20 (16.67%) | 14 (11.67%) | 65 (54.17%) | 16 (13.33%) |  |
| 23 | Vigorous-intensity | 20 (16.67%) | 46 (38.33%) | 19 (15.83%) | 28 (23.33%) | 7 (5.83%) |  |
| 24 | Keep fast | 34 (28.33%) | 56 (46.67%) | 15 (12.5%) | 14 (11.67%) | 1 (1.83%) |  |
| 25 | Skip meals | 31 (25.83%) | 56 (46.67%) | 9 (7.5%) | 21 (17.5%) | 3 (2.5%) |  |
| 26 | Drink water | 9 (7.5%) | 24 (20%) | 11 (9.17%) | 56 (46.67%) | 20 (16.67%) |  |
| 27 | Low-fat meals | 5 (4.17%) | 26 (21.67%) | 10 (8.33%) | 63 (52.5%) | 16 (13.33%) |  |
| 28 | Low-carbohydrate meals | 4 (3.33%) | 38 (31.67%) | 12 (10%) | 53 (44.17%) | 13 (10.83%) |  |
| 29 | High-fibre foods | 4 (3.33%) | 28 (23.33%) | 20 (16.67%) | 56 (46.67%) | 12 (10%) |  |
| 30 | Portion sizes | 7 (5.83%) | 26 (21.67%) | 17 (14.17%) | 60 (50%) | 10 (8.33%) |  |
| 31 | Nutritional labels | 14 (11.67%) | 33 (27.5%) | 21 (17.5%) | 42 (35%) | 10 (8.33%) |  |
| 32 | Monitor consumption | 6 (5%) | 24 (20%) | 18 (15%) | 57 (47.5%) | 15 (12.5%) |  |
| 33 | Avoid eating outside | 1 (0.83%) | 32 (26.67%) | 13 (10.83%) | 51 (42.5%) | 23 (19.17%) |  |
| 34 | Limit consumption | 2 (1.67%) | 29 (24.17%) | 15 (12.5%) | 60 (50%) | 14 (11.67%) |  |
| 35 | Monitor activity level | 12 (10%) | 52 (43.33%) | 23 (19.17%) | 31 (25.83%) | 2 (1.67%) |  |
| 36 | Liquid meal replacement | 17 (14.17%) | 60 (50%) | 16 (13.33%) | 22 (18.33%) | 5 (4.17%) |  |
| 37 | Track | 20 (16.67%) | 54 (45%) | 19 (15.83%) | 25 (20.83%) | 2 (1.67%) |  |
| 38 | Amount of calories | 18 (15%) | 55 (45.83%) | 23 (19.17%) | 21 (17.5%) | 3 (2.5%) |  |
| 39 | Minimise sedentary activity | 10 (8.33%) | 46 (38.33%) | 18 (15%) | 40 (33.33%) | 6 (5%) |  |
| 40 | Stairs | 11 (9.17%) | 51 (42.5%) | 16 (13.33%) | 34 (28.33%) | 8 (6.67%) |  |
|  |  | **Strongly disagree** | **Disagree** | **Neither** | **Agree** | **Strongly agree** |  |
| 41 | Health professional | 21 (17.5%) | 58 (48.33%) | 20 (16.67%) | 20 (16.67%) | 1 (0.83%) |  |
| 42 | Separate meals | 24 (20%) | 47 (39.17%) | 18 (15%) | 27 (22.5%) | 4 (3.33%) |  |
| 43 | Measuring portion sizes | 21 (17.5%) | 38 (31.67%) | 20 (16.67%) | 38 (31.67%) | 3 (2.5%) |  |
| 44 | Physical activities | 19 (15.83%) | 41 (34.17%) | 16 (13.33%) | 39 (32.5%) | 5 (4.17%) |  |
| 45 | Income | 2 (1.71%) | 15 (12.82%) | 23 (19.66%) | 63 (53.85%) | 14 (11.97%) | 3 |
|  |  |  |  |  |  |  |  |
